# Environmental Health Burdens and Socioeconomic Status in Rhode Island: Using Geographic Information Systems to Examine Health Disparities in Medical School

**DOI:** 10.7759/cureus.9816

**Published:** 2020-08-17

**Authors:** King John Pascual, Andrew Palosaari, Jacqueline Ochoa, Claudia Dreyer

**Affiliations:** 1 Department of Medicine, George Washington University School of Medicine and Health Sciences, Washington, D.C., USA; 2 Department of Public Health, Tufts University School of Medicine, Boston, USA; 3 Department of Biological Sciences, George Washington University, Washington, D.C., USA

**Keywords:** gis, medical geography, public and environmental health, structural racism, rhode island

## Abstract

Race and class are major predictors of health outcomes in the United States. Health disparities among racial and low-income minorities often have environmental etiologies. Using Rhode Island as a case study, we geocoded and visualized several environmental determinants of health via Geographic Information Systems (GIS) in the entire state and conducted a geospatial analysis to determine whether or not patterns existed along racial and class lines. The variables that we geocoded include elementary schools, fast food restaurants, Superfund sites, and community parks. From a census tract level, we then analyzed the racial and income makeup of each geocoded site. We discovered that, on average, the worst-performing elementary schools, fast food restaurants, and Superfund sites in Rhode Island were clustered in neighborhoods with a larger black population and lower household income. Conversely, community parks and the best elementary schools in Rhode Island tended to be located near neighborhoods with a larger White population and higher household income. Our results provide additional evidence for the pervasiveness of the unequal distribution of environmental health burdens between low-income, minority communities and affluent, predominantly White communities. This summer experiential student project demonstrates the feasibility of incorporating GIS as a practical tool for learning health disparities material at a U.S. medical school. Our study also highlights the value of digital technology and citizen science in helping the public recognize and understand the various environmental factors that perpetuate health disparities.

## Introduction

The evidence in the scientific literature documenting the prevalence of racial and ethnic health disparities in the United States is profound. And yet between 1999 and 2011, we only saw a modest increase in public awareness on the prevalence of health disparities [1]. Moreover, there seems to be public resistance to the notion that health inequality is heavily influenced by social and environmental factors. Many Americans believe that health disparities that affect low-income and minority communities are borne out primarily by personal choices, as opposed to structural and historical factors [2,3]. Physician awareness and attitudes towards health disparities have also been a subject of concern. Many physicians report not having sufficient knowledge and context of their patients’ communities and the specific health barriers they face [4]. While the majority of medical schools today include health disparities in their curriculum, many report that they do not feel satisfied with the quality of course offerings [5]. Furthermore, health disparities material, if not implemented well into medical school curriculum, can be met with negative attitudes by medical students [6].

Geography can help us understand and contextualize health and medicine in many ways. By having access to geographical data, we can analyze the onset, duration, and aftermath of diseases, which can, in turn, help public health advocates and policymakers create interventions that improve the health outcomes of their constituents. It should be no surprise then that “medical geography” has become a burgeoning area of research [7].

In this article, we use Geographic Information Systems (GIS) to visualize the distribution of environmental health burdens in Rhode Island. Specifically, we wanted to see if fast-food restaurants, Superfund sites, failing schools, and community parks clustered in certain neighborhoods. We used Rhode Island as a case study due to its small size and the feasibility to conduct a statewide geospatial analysis. The limited number of health disparities research on Rhode Island also compelled us to use it for our study. Our goal was to offer visual and concrete evidence of whether or not environmental health burdens disproportionately impact low-income and minority communities. We utilized a digital tool (i.e. GIS) that we believe has the potential to increase awareness of health disparities among healthcare professionals and the public at large. Our study aims to continue the national discourse on health disparities by helping the public recognize how social and environmental burdens exist along class and racial lines. We hope that our findings could serve as an additional resource for public health advocates and policymakers, both in Rhode Island and around the country.

Geography and social inequality

Geography serves as a medium where inequalities “may be passed on from one generation to the next via the environment of opportunities and services into which an individual is implanted at birth” [8]. Historically in the United States, low-income and racial minority communities have been subject to systematic discrimination. Jim Crow laws prohibited black students from attending schools with their peers. Redlining has deprived communities of color generational wealth that their White counterparts now benefit from [9]. Decades of predatory marketing and advertisements by fast-food corporations such as McDonald’s and KFC have contributed to the obesity epidemic in black families [10]. More recently, national discussions spearheaded by African-American communities in Flint, Michigan and Native Americans in North Dakota bear witness to the ongoing issue of environmental injustice that predominantly affects low-income and racial minorities [11,12]. Because geography has played an inherent role in the development and perpetuation of social inequality, GIS can therefore be a practical tool for examining the intersections of socioeconomic status and environmental health burdens.

GIS and citizen science

Geographic Information Systems is a visual and analytical tool with a multitude of applications. Its users widely range from journalists and activists to marketing and advertising agencies [13-15]. GIS has been used to track human rights violations in Uganda, document changes in population across the United States by race and ethnicity, and show locations of deforestations and mining concessions throughout the Amazon rainforest in real-time [16-18]. In public health research, the most common GIS applications include disease surveillance, risk analysis, health access planning, and community health profiling [19].

Citizen science can be broadly defined as “the general public engagement in scientific research activities when citizens actively contribute to science either with their intellectual effort or surrounding knowledge or with their tools and resources” [20]. Central to citizen science is the mission to democratize data. Initially, citizen science was predominantly seen in the natural sciences field but has been gradually embraced as a method of data collection in other fields such as economics, journalism, and political science. However, citizen science in public health can be argued as rare and didn’t gain worldwide traction until the recent COVID-19 pandemic. In this study, we utilize a multi-disciplinary approach that combines GIS and citizen science to examine the distribution of environmental health burdens in Rhode Island using data from publicly available sources.

## Materials and methods

Addresses for local branches of KFC (n = 17), McDonald’s (n = 16), and Taco Bell (n = 5) in Rhode Island were retrieved using company websites. Locations of the top 30 and bottom 30 elementary schools in the state were collected based on data from www.schooldigger.com. Public parks (n = 30) with community playgrounds were identified from www.rifamilyguide.com. To identify facilities that release toxic substances and pollutants in Rhode Island, the U.S. Environmental Protection Agency’s Envirofacts database was used, from which 30 sites were randomly chosen for the study sample. The retrieved addresses were compiled in Microsoft Excel and geo-coded in ArcMap using the 1983 NSRS2007 StatePlane coordinate system for Rhode Island. Addresses that were not successfully geocoded initially and those that did not have a full street address were replaced by the closest address/intersection. A spatial join was then conducted between the geocoded point features and maps of census tracts containing American Community Survey (ACS) data which was used to determine percent distribution of White population and average household income in surrounding neighborhoods. Pearson correlations and independent sample t-tests were also used for analysis.

## Results

Education

**Figure 1:**
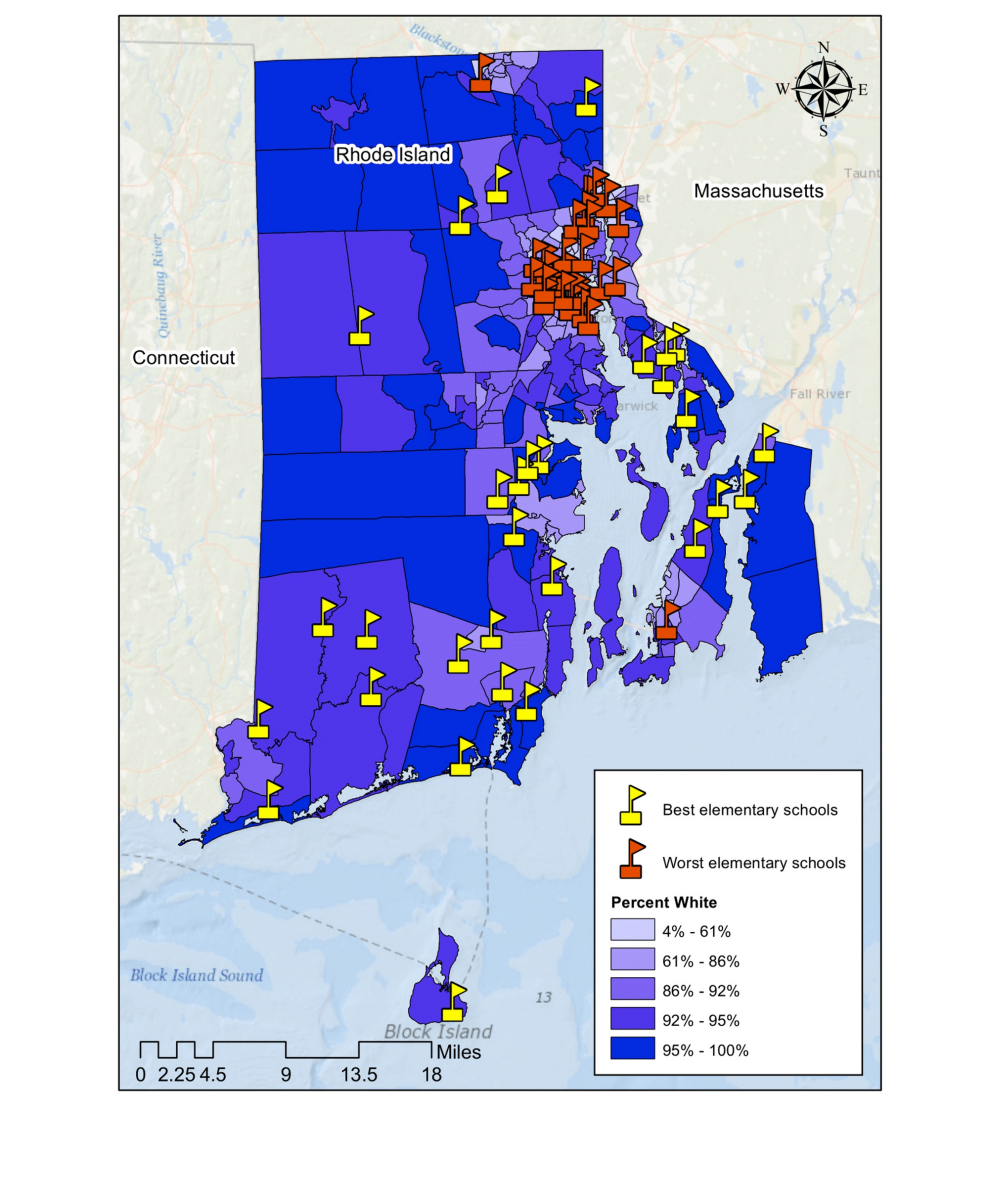
Geographic distribution of the top and bottom ranked elementary schools in Rhode Island

The neighborhoods surrounding the bottom-ranked elementary schools had a lower average household income and percentage of White population than those surrounding the top-performing elementary schools in Rhode Island (Table 1). The difference was statistically significant with a p-value of less than 0.0001 (α = 0.05). Furthermore, the bottom-ranked elementary schools tended to be clustered in the Providence County area compared to the top-performing elementary schools (Figure 1). 

**Table 1:** Average income and percent white population of neighborhoods surrounding the top-ranked and bottom-ranked elementary schools in Rhode Island

	Top-ranked schools (n=30)	Bottom-ranked schools (n=30)	P-value
Avg. income of closest census tract	$79,159.63	$36,408.28	<0.0001
Standard deviation of avg. income	$24,597.56	$9,152.62	
Avg. % of white population	$92.8%	36.4%	<0.0001
Standard deviation of avg. %	3.53%	25.0%	

Community parks

**Figure 2:**
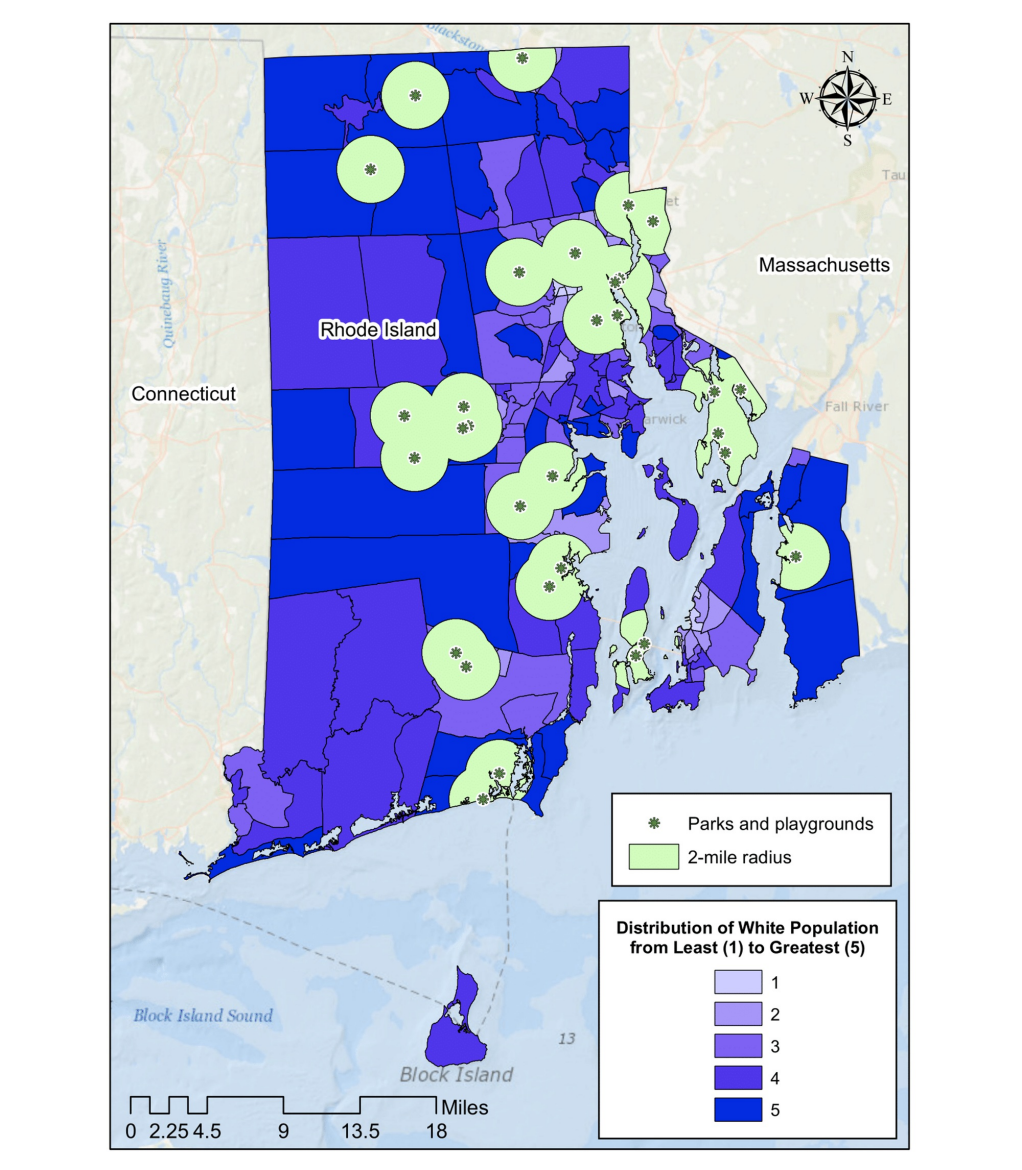
Geographic distribution of community parks and playgrounds showing a two-mile radius catchment area

**Figure 3:**
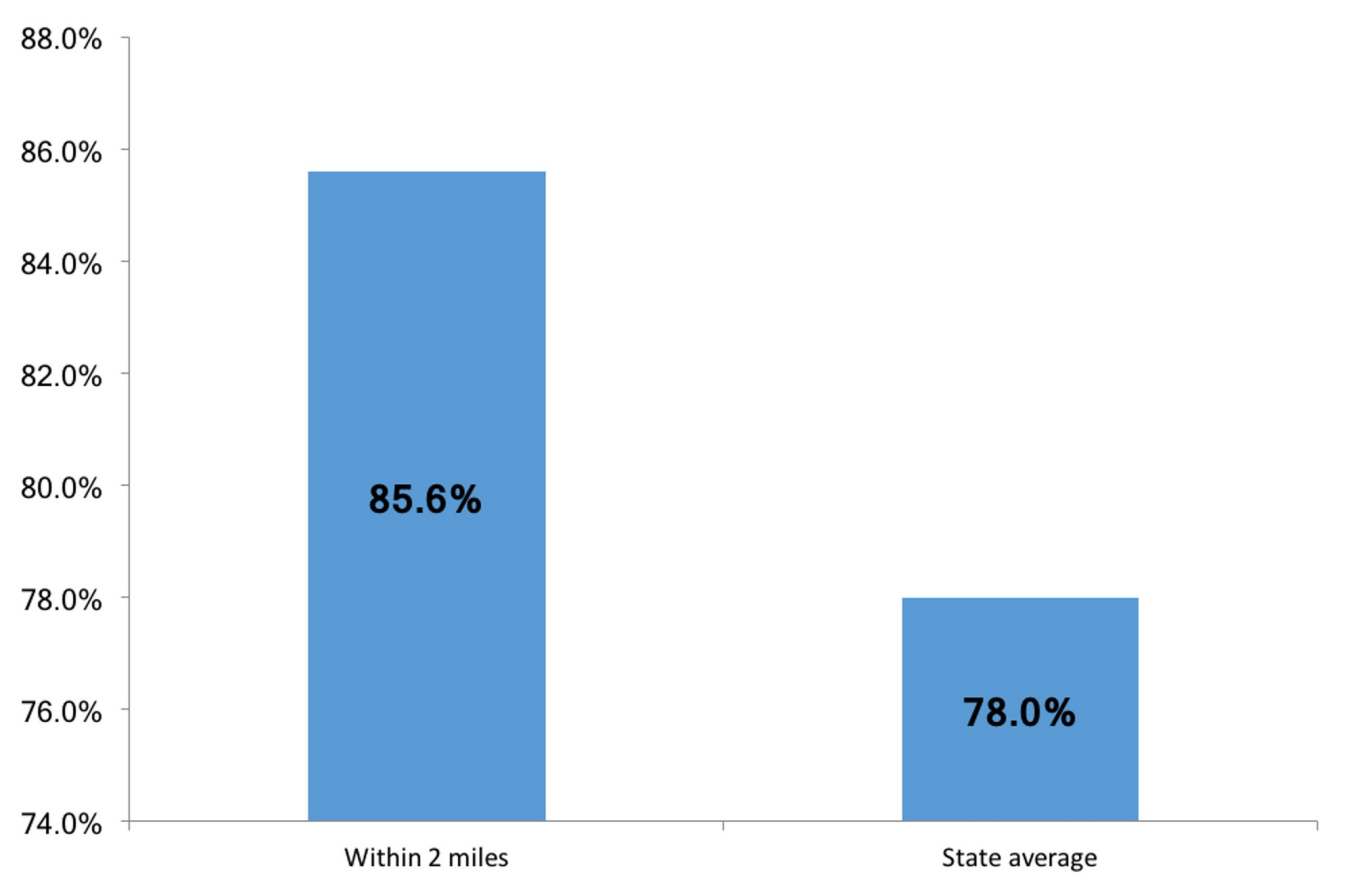
Percent of White population in neighborhoods within two miles of a community park compared to the state average

**Figure 4:**
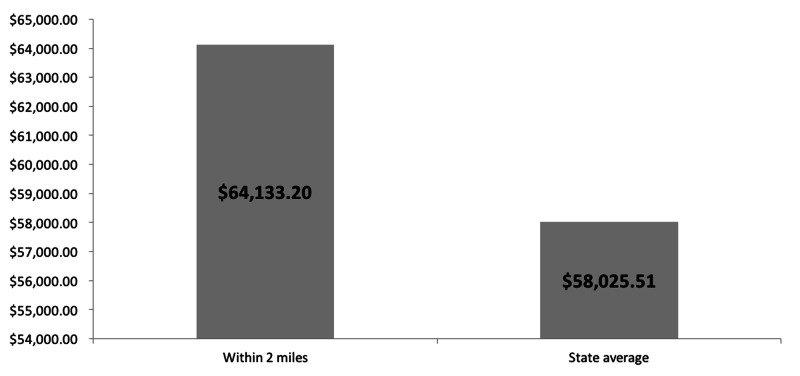
Average household income of census tracts within two miles of a community park compared to the state average household income

Census tract neighborhoods within a two-mile radius of community playgrounds (Figure 2) were 85.6% White and had an average income of $64,133.20 (Figures 3, 4), which were 7.6% and $6,107.69 higher than the state average, respectively. The differences however were not statistically significant (p-value=0.16 and p-value=0.91). 

Fast-food restaurants and Superfund sites

**Figure 5:**
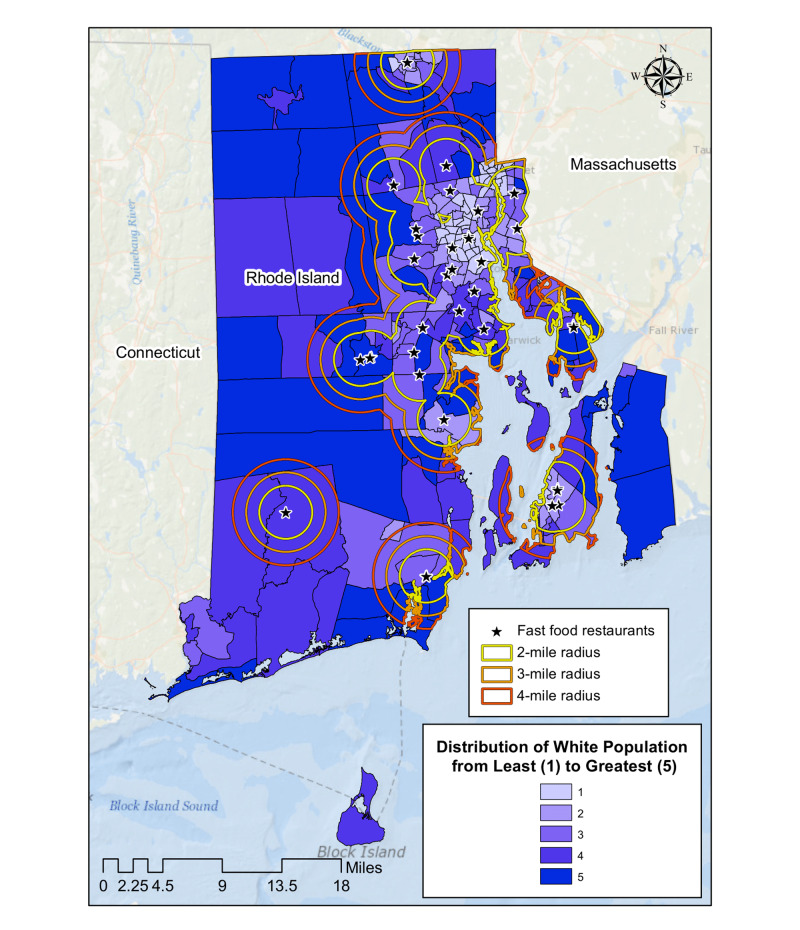
Geographic distribution of three major fast food restaurants in Rhode Island

**Figure 6:**
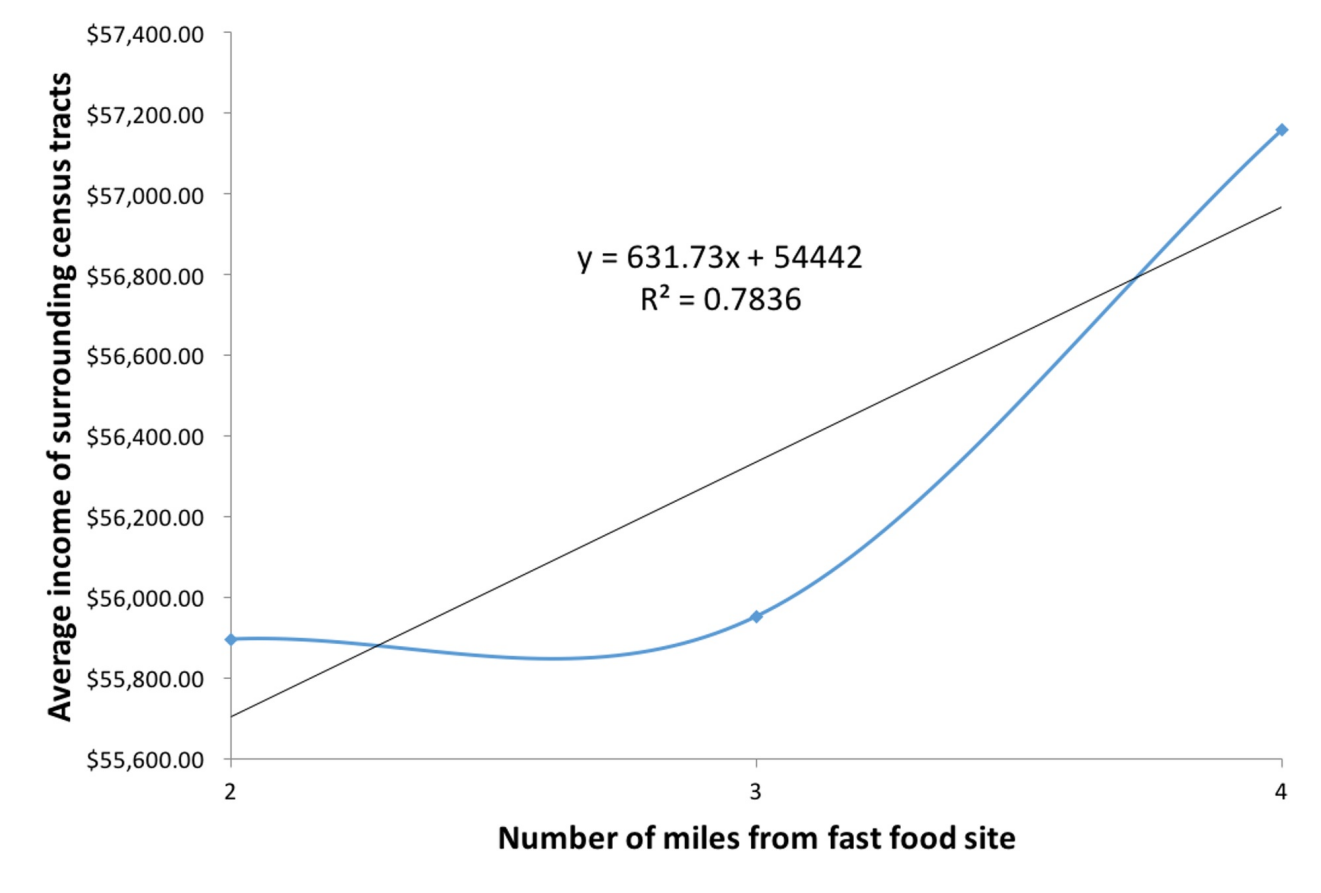
Average income of census tracts surrounding geocoded fast food restaurants

**Figure 7:**
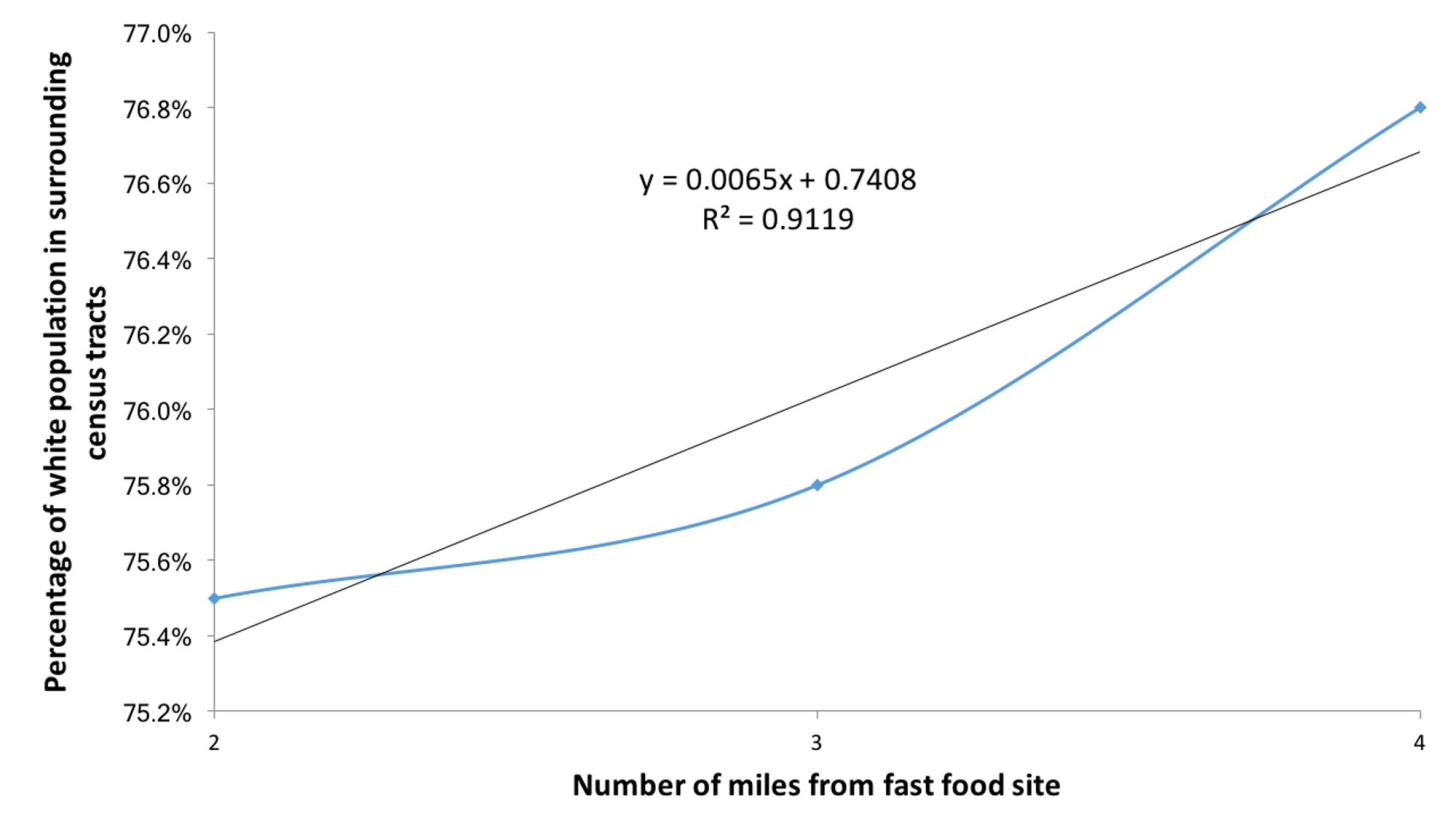
Average percentage of white population in census tracts surrounding geocoded fast food restaurants

Fast food restaurants such as KFC, McDonald’s, and Taco Bell also tended to be located in neighborhoods with a smaller percentage of White population (Figure 5). Both census tract values for average household income and percentage of White population increase as the distance from the fast-food site increases, with correlation coefficients of r(3)=0.89 and r(3)=0.95, respectively (Figures 6, 7).

**Figure 8:**
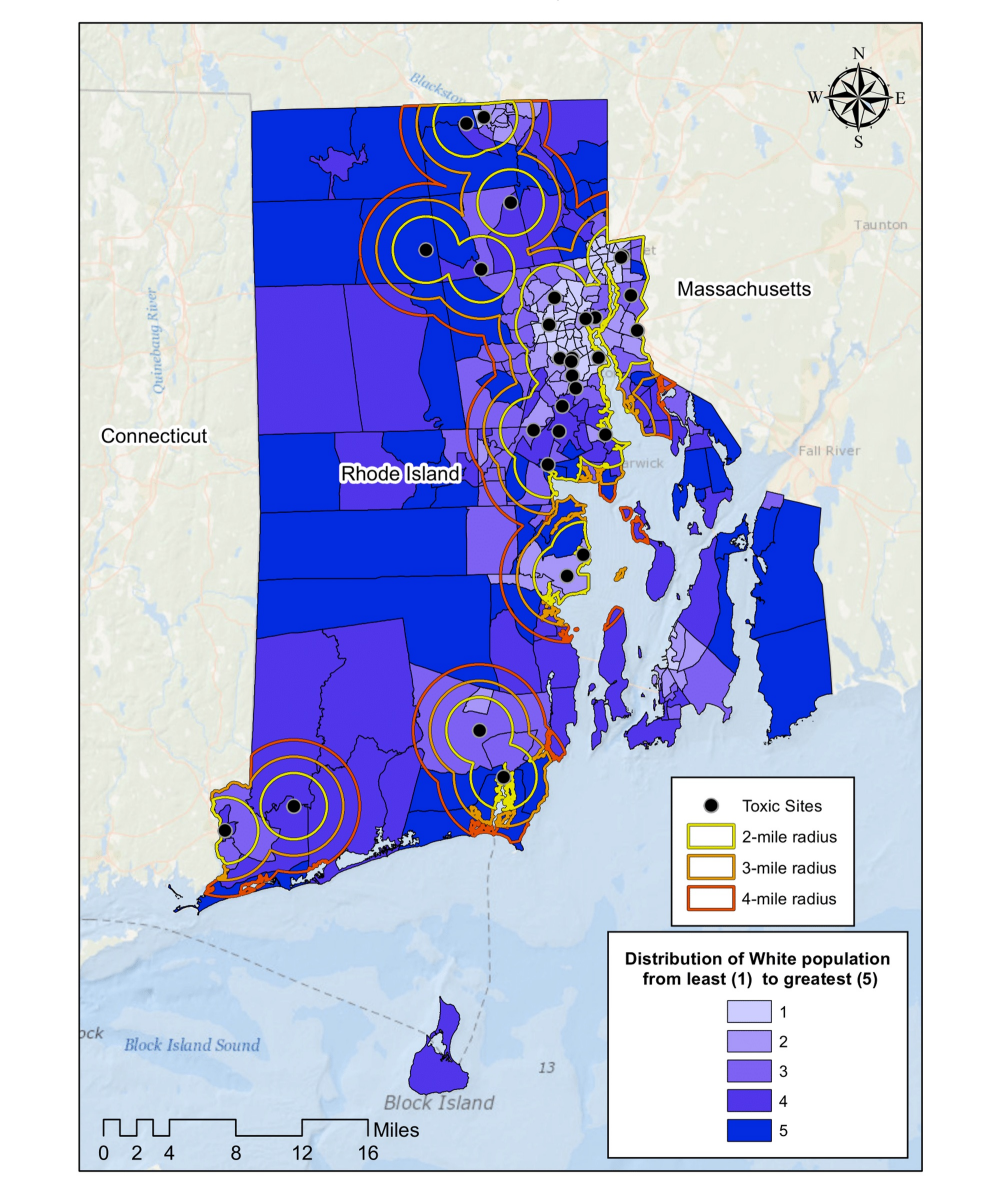
Geographic distribution of 30 randomly chosen Superfund sites in Rhode Island

**Figure 9:**
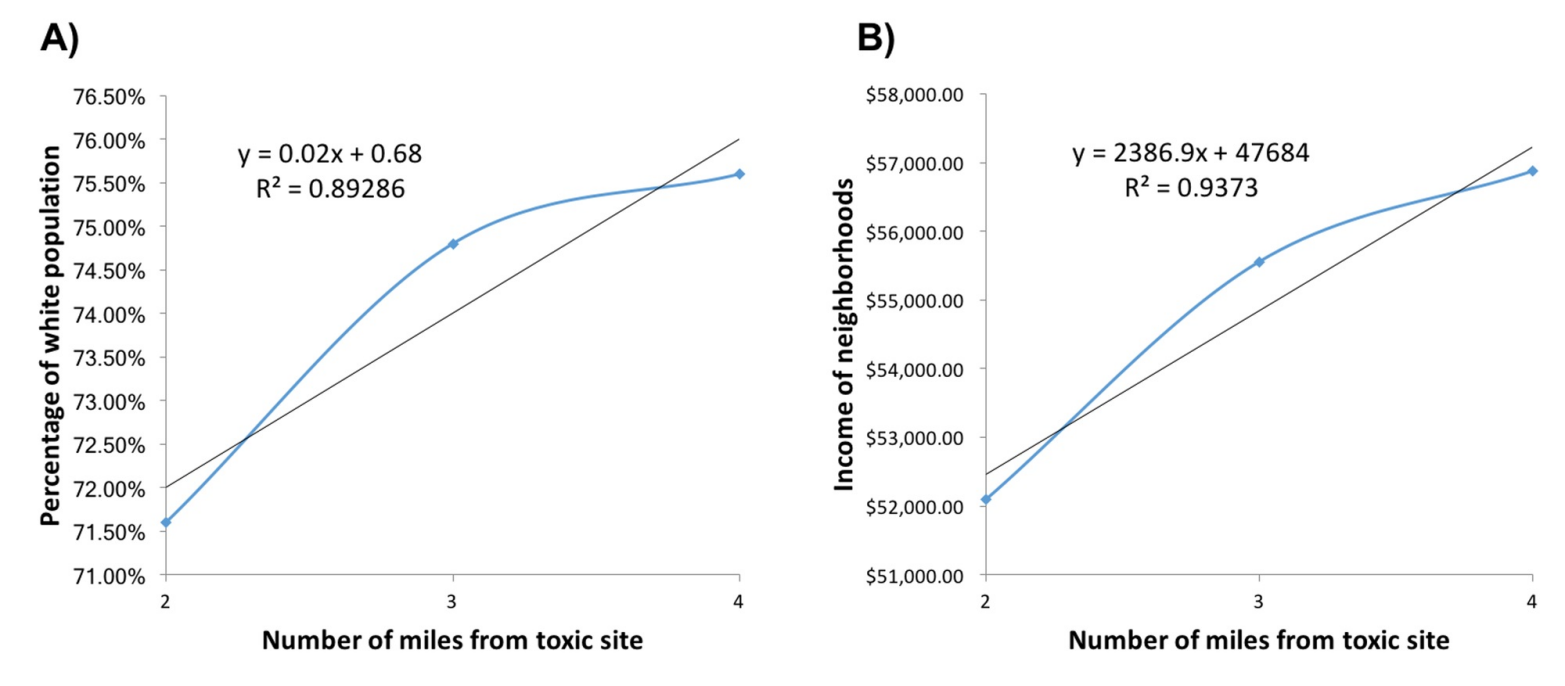
Socioeconomic makeup of neighborhoods surrounding Superfund sites in Rhode Island A) Average percentage white population in surrounding census tracts as a function of distance from Superfund sites B) Average household income of surrounding census tracts as a function of distance from Superfund sites

Figure 8 shows the locations of 30 randomly chosen Superfund sites in Rhode Island. The term Superfund is used to describe the hazardous waste sites that have been designated by the government for long-term cleanup. We geocoded 30 of these sites across Rhode Island and measured the racial and class make-up of neighborhoods within a two, three, and four-mile radius. Our results reveal a positive linear relationship between distance from toxic sites and % White population [r(3)=0.94] as well as average household income [r(3)=0.97] of surrounding census tracts (Figure 9).

**Figure 10:**
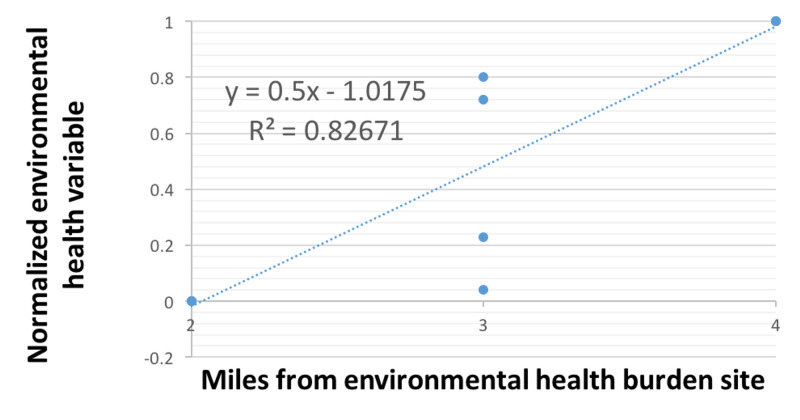
Normalized index of average household income and percent white population as a function of distance from geocoded Superfund and fast food sites

We also performed a test for the significance of the correlation coefficient between neighborhood distance and the measured values for fast-food restaurant and Superfund sites [r(12)=0.91, p<0.001]. To address the different units of measurement, average household income and % White population were both normalized for this analysis (Figure 10).

## Discussion

This study was primarily conducted by U.S. medical students as a summer experiential project to learn about health disparities and medical geography using a practical data analytics tool (i.e. Geographic Information Systems). Our findings reinforce the notion that lack of access to nutritious foods, multiple sources of environmental pollution, poor quality education, and lack of access to community parks disproportionately impact communities of color. For our first geospatial analysis, we specifically showed that the top-ranked elementary schools were mostly located in affluent and largely White neighborhoods, while the worst-ranked elementary schools were clustered in low-income and largely Black neighborhoods. The associations were statistically significant (p<0.0001). This finding is concerning because quality of early childhood education has been shown to be a reliable predictor of health outcomes. Students who attend high-quality schools, which often have comprehensive science and health curricula, have been shown to demonstrate higher levels of health literacy as adults [21]. They are also more likely to be smarter consumers of medical information as well as stronger advocates of their own health when interacting with their physicians [22].

We demonstrated the same pattern in race and class makeup of neighborhoods when looking at placements of community parks in Rhode Island. The communities that are within two miles of parks had higher household incomes and larger percentage of White residents compared to the state average. Research shows that availability of green spaces has been linked to improved mental health and reduction in cardiovascular risk [23]. Considering the disproportionately high rates of chronic stress and diabetes in Black and low-income patients, our data underscores the need for Rhode Island city planners to increase access to green spaces and community parks in low-income, minority neighborhoods. 

Our subsequent geospatial analysis revealed associations between socioeconomic makeup of neighborhoods and placement of fast-food restaurants. On average, we discovered that household income increases as one travels farther from the address of a fast-food restaurant. While we did not directly examine the prevalence of food deserts, this finding aligns with the numerous studies documenting the high prevalence fast-food restaurants in Black neighborhoods, which may help explain the obesity epidemic in these communities. In a geographical survey study, Beaulac et al. documented that in areas with a high proportion of low-income or African American residents, there are fewer supermarkets or chain stores per capita when compared to more economically advantaged areas [24]. The authors concluded that low-income, minority areas tend to have poor access to healthy food. Studies also suggest that supermarkets may be intentionally avoiding such neighborhoods because of the lower purchasing power in these communities [25]. Perceptions that high crime and cultural norms will significantly hinder profit margins of supermarkets could be additional factors. That fast food is ingrained in the diet of African-Americans can also be attributed to the intentional targeting of Black communities by fast-food company advertisers [10].

Lastly, we conducted a geospatial analysis of Superfund site locations and found that the neighborhoods in close proximity also tended to be more Black and less affluent. This was not a surprising finding due to the well-documented historical data on the strategic placement of toxic facilities in minority and low-income neighborhoods [26]. Dating back to the 1960s, politicians and urban planners have historically avoided White and affluent neighborhoods when planning for the construction of hazardous waste facilities such as landfills, industrial factories, and incinerators. One aim of our study is to offer yet another framework through which health disparities and environmental racism can be conceptualized.

This study is the first to utilize GIS to examine the relationship between community demographic data and the distribution of multiple sources of environmental health burden in Rhode Island. Popick et al. used Rhode Island as a GIS case study but only focused on access to care through proximity to bus lines and physician distribution by region and per capita [27]. Dong et al. also examined Rhode Island using GIS as a tool [28]. However, their focus was on food insecurity among adults who are on probation. Other health-related GIS studies on Rhode Island have involved an evaluation of flood-prone areas, nitrogen dioxide levels, and neighborhood walkability [29,30].

Our study has limitations. First, we did not examine specific health conditions in Rhode Island. Exploring the correlations between environmental health burdens and the prevalence of relevant illnesses like asthma and cardiovascular disease could lend more weight to the argument that GIS research is critical for population health and can serve as a resource for health policy officials. Second, our census tract dataset did not include Asians, Hispanics, and Native-Americans. Future research on GIS and health disparities, in general, should define “minority communities” in a way that it includes all communities of color. One unique strength in our study was the decision to use publicly available data (e.g. EPA website, fast food online directories, schooldigger.com, Google Maps, rifamilyguide.com). Doing so demonstrates the feasibility and value of conducting “citizen science” research, a growing field of interest in our current era of big-data. In addition to reaffirming the value of GIS for urban planning and community health, we also believe that our study has practical implications for medical education and the national discourse on health disparities.

As we mentioned earlier in this paper, it seems that a contingent of Americans is resistant to the notion that health outcomes are significantly influenced by social and environmental forces that are historically rooted in classism and racism. Moreover, physicians may be ill-equipped to provide culturally competent care to their patients due to their lack of knowledge or misperceptions about the fundamental causes of health disparities. Given the death rate from the 2019 novel coronavirus among African-American and low-income patients, it should be no surprise if environmental racism and medical geography resurface as major topics of concern for American public health leaders and politicians. We thus recommend that digital platforms such as GIS be used to visualize health disparities data in an accessible and interactive way. Doing so may increase the public’s awareness and level of engagement to learn more about the social and environmental factors that determine health outcomes and perhaps even spearhead citizen science projects themselves. We would also argue that GIS combined with citizen science can be an engaging, innovative, and experiential modality to teach health disparities in medical school. If, for example, medical students are offered an opportunity to learn GIS and practice citizen science, they would be better equipped to become public health advocates. Not to mention, they will gain a technical skillset critical in a field that is relying more and more on healthcare innovation to improve patient outcomes on a population level.

## Conclusions

The data suggest that the locations of schools, fast food restaurants, Superfund sites, and community parks are associated with the racial and economic makeup of their surrounding census tract neighborhoods. Worst performing elementary schools, fast food restaurants, and toxic facilities were more likely to be surrounded by poorer and less White neighborhoods. In contrast, the best performing elementary schools and community parks were more likely to be placed in affluent and predominantly White neighborhoods. These findings are consistent with the literature on the relationship between systemic discrimination and health disparities. The results in this study may also help our current understanding of health disparities in Rhode Island and the United States as a whole. Future studies should explore the link between specific health outcomes and environmental health burdens in Rhode Island. When defining “minority communities,” studies should include Asian, Hispanic, and Native American neighborhoods. Identification of other potential etiological factors to health disparities should also be a subject of future GIS research. Lastly, incorporation of GIS into health disparities curriculum or summer experiential projects during medical school presents a practical and engaging learning opportunity to conceptualize the racial, economic, and environmental determinants of health in the United States.

## References

[1] Benz JK, Espinosa O, Welsh V (2011). Awareness of racial and ethnic health disparities has improved only modestly over a decade. Health Affairs.

[2] Gollust SE, Cappella JN (2014). Understanding public resistance to messages about health disparities. J Health Commun.

[3] Bye L, Ghirardelli A, Fontes A (2016). Promoting health equity and population health: how Americans’ views differ. Health Affairs.

[4] Nesbitt S, Palomarez RE (2016). Review: Increasing awareness and education on health disparities for health care providers. Ethn Dis.

[5] Chen FM, Overstreet F, Cole A, Kost A, Brown Speights JS (2017). Racial and ethnic health disparities curricula in US medical schools: A CERA study. PRiMER.

[6] Gonzalez CM, Bussey-Jones J (2010). Disparities education: what do students want?. J Gen Intern Med.

[7] Musa GJ, Chiang P, Sylk T (2013). Use of GIS mapping as a public health tool-from cholera to cancer. Health Serv Insights.

[8] Peet R (1975). Inequality and poverty: a Marxist-geographic theory. Ann Assoc Am Geogr.

[9] Killewald A, Pfeffer FT, Schachner JN (2017). Wealth inequality and accumulation. Annu Rev Sociol.

[10] Grier SA, Kumanyika SK (2008). The context for choice: health implications of targeted food and beverage marketing to African Americans. Am J Pub Health.

[11] Campbell C, Greenberg R, Mankikar D (2016). A case study of environmental injustice: the failure in Flint. Int J Environ Res Public Health.

[12] Lewis J, Hoover J, MacKenzie D (2017). Mining and environmental health disparities in Native American communities. Curr Environ Health Rep.

[13] Wasike BS (2005). The diffusion of GIS in journalism. https://digitalcommons.lsu.edu/gradschool_dissertations/2643/.

[14] O’Rourke MJ (2018). The map is not the territory: applying qualitative geographic information systems in the practice of activist archaeology. J Social Archaeology.

[15] Viswanathan NK (2005). GIS in marketing. Geographic Information Systems in Business.

[16] Madden M, Ross A (2009). Genocide and GIScience: integrating personal narratives and geographic information science to study human rights. The Professional Geographer.

[17] Reibel M (2007). Geographic information systems and spatial data processing in demography: a review. Popul Res Policy Rev.

[18] Gutierrez M (2019). Maputopias: cartographies of communication, coordination and action—the cases of Ushahidi and InfoAmazonia. GeoJournal.

[19] Fletcher-Lartey SM, Caprarelli G (2016). Application of GIS technology in public health: successes and challenges. Parasitology.

[20] Socientize Consortium (2013). Green paper on citizen science. Citizen Science for Europe. Towards a better society of empowered citizens and enhanced research. Brussels. https://ec.europa.eu/digital-single-market/en/news/green-paper-citizen-science-europe-towards-society-empowered-citizens-and-enhanced-research.

[21] Jacque B, Koch-Weser S, Faux R (2016). Addressing health literacy challenges with a cutting-edge infectious disease curriculum for the high school biology classroom. Health Educ Behavior.

[22] Winkelman TN, Caldwell MT, Bertram B (2016). Promoting health literacy for children and adolescents. Pediatrics.

[23] Dzhambov AM (2018). Residential green and blue space associated with better mental health: a pilot follow-up study in university students. Arh Hig Rada Toksikol.

[24] Beaulac J, Kristjansson E, Cummins S (2009). A systematic review of food deserts, 1966-2007. Prev Chronic Dis.

[25] Ohri-Vachaspati P, DeWeese RS, Acciai F (2019). Healthy food access in low-income high-minority communities: a longitudinal assessment-2009-2017. Int J Environ Res Public Health.

[26] O’Neil SG (2007). Superfund: Evaluating the impact of executive order 12898. Environ Health Perspect.

[27] Popick R, Arthur Frazzano M, Trachtenberg R (2009). Geographic access to care in Rhode Island through the use of GIS. Med Health R I.

[28] Dong KR, Tang AM, Stopka TJ (2018). Food acquisition methods and correlates of food insecurity in adults on probation in Rhode Island. PloS One.

[29] Hardmeyer K, Spencer MA (2007). Using risk-based analysis and geographic information systems to assess flooding problems in an urban watershed in Rhode Island. Environ Manage.

[30] Carr LJ, Dunsiger SI, Marcus BH (2010). Walk score™ as a global estimate of neighborhood walkability. Am J Prev Med.

